# Sexual risk-taking behavior amongst emerging adults in a tertiary institution of learning in Coastal Kenya: A qualitative study of stakeholders’ perspectives using causal loop mapping

**DOI:** 10.1371/journal.pone.0284550

**Published:** 2023-10-10

**Authors:** Stevenson K. Chea, Vincent A. Kagonya, Eunice A. Oyugi, Carophine Nasambu, Isaac Menza, Fauz Ibrahim, Osman Abdullahi, Alice Anika, Amin S. Hassan, Souheila Abbeddou, Kristien Michielsen, Amina Abubakar

**Affiliations:** 1 Department of Nursing Sciences, School of Health and Human Sciences, Pwani University, Kilifi, Kenya; 2 Faculty of Medicine and Health Sciences, Department of Public Health and Primary Care, International Centre for Reproductive Health, Ghent University, Ghent, Belgium; 3 Faculty of Medicine and Health Sciences, Department of Public Health and Primary Care, Public Health Nutrition Unit, Ghent University, Ghent, Belgium; 4 Centre for Geographic Medicine Research (Coast), Kenya Medical Research Institute/Wellcome Trust Research Programme, Kilifi, Kenya; 5 County Department of Health, Kilifi, Kenya; 6 Department of Public Health, School of Health and Human Sciences, Pwani University, Kilifi, Kenya; 7 Department of Educational Psychology, School of Education, Pwani University, Kilifi, Kenya; 8 Institute for Human Development, The Aga Khan University, Nairobi, Kenya; University of Nairobi, KENYA

## Abstract

**Background:**

It is known from previous studies that university students in sub-Saharan Africa (sSA) engage in sexual risk-taking behaviour (SRTB). However, there is paucity of data on factors contributing to SRTB among university students (emerging adults) at the Kenyan Coast thus hindering intervention planning. This study seeks to provide an in-depth qualitative understanding of the factors contributing to SRTB and their interconnectedness among university students at the Kenyan Coast combining qualitative research with a systems thinking approach.

**Methods:**

Using the ecological model, and employing in-depth interviews, we explored the perceptions of twenty-six key informants (twenty-one emerging adults and five other stakeholders) on what constitutes and influences SRTB among emerging adults at a tertiary institution of learning in Coastal Kenya. Data were analysed using a thematic framework approach. A causal loop diagram (CLD) was developed to map the interconnectedness of the correlates of SRTB.

**Results:**

Our findings show that unprotected sex, transactional sex, cross-generational sex, multiple sex partnerships, gender-based violence, sex under influence of alcohol/drugs, early sex debut, and sharing sex toys were common SRTBs. Based on the ecological model and CLD, most of the reported risk factors were interconnected and operated at the individual level.

**Conclusion:**

Our study shows that emerging adults are frequently engaging in unprotected sex. Enhancing sexuality education programs for students in Kenyan universities and strengthening support systems including counselling for those using alcohol/drugs may help reduce SRTB among emerging adults in Kenyan universities.

## Background

Sexual risk-taking behaviour (SRTB) including non-condom use [1–4], concurrent sexual partners [5,6], multiple sexual partnerships [1,5,7–11], early sex debut [5,10–12], age-disparate relationships [3,7,9] and transactional sex [1,3,6,11,13] is well documented among young people in sub-Saharan Africa (sSA) and remains common [14]. Accordingly, sSA bears the greatest burden of HIV infection among young people [15], with young women accounting for one in four new infections in 2019, despite making up only 10% of the total population [15]. Although progress has been made towards scaling down the HIV pandemic, Kenya remains one of the high burden countries in sSA [16]. In 2019, a total of 41,408 people were newly infected with HIV in Kenya, with 15–29 year old contributing 62% of all new infections [16].

Emerging adulthood is a developmental period from the late teens through the twenties, with a focus on ages 18–25 [17]. Emerging adults’ brains are still developing [18], which increases risk for sub-optimal performance on executive function with a heightened propensity for engaging in SRTB as a consequence [18]. Further, sex tourism and drug abuse reported at the Kenyan Coast put emerging adults in this region at a higher risk of SRTB [19,20]. According to the ecological model, risk and protective factors of SRTB fall into six domains: i) macro, ii) social, iii) school, iv) family, v) peers, and vi) individual [21]. Additionally, there has been less attention to the causal mechanisms underpinning the occurrence of SRTB among emerging adults. Systems thinking enables understanding of inter-relationships and interactions within a system [22]. Causal loop diagrams (CLDs) are a systems thinking method that can be used to unpack complex health system behaviour [23]. As a methodology, the current study utilizes a causal loop diagram to show the connectedness of factors contributing to SRTB therefore enhancing their understanding.

Exploring the experiences of emerging adults and opinions of other stakeholders on SRTB is important in designing targeted interventions. Some of the factors underlying SRTB explored in previous studies include socio-demographic and relationship factors [24–28]. A Kenyan study conducted among university of Nairobi students explored correlates of SRTB [28]. The University of Nairobi study is quantitative therefore, limiting understanding of the mechanisms underlying the drivers reported from the participants point of view.

Altogether, it is known from previous studies [10,25–46] that university students in Sub-Saharan Africa engage in SRTB. However, there is limited in-depth qualitative understanding of the factors contributing to SRTB and their connectedness among university students at the Kenyan Coast. Understanding the factors contributing to SRTB and their connectedness helps in prioritizing interventions. This study seeks to provide an in-depth qualitative understanding of SRTB among university students at the Kenyan Coast using a systems thinking approach.

## Methods

### Study design

A qualitative study incorporating systems thinking was conducted at Pwani university in Coastal Kenya between October 31^st^ 2019 and March 16^th^ 2020. Pwani university has an estimated population of 8000 students [47] out of whom about 98% are regular undergraduate students. Key informants included undergraduate students aged 18–24 years and other stakeholders including the Dean of students, Student Counsellor, and nurses working at the students’ health unit and the university HIV voluntary counselling and testing centre (VCT).

### Recruitment

Recruitment of undergraduate students was through snowballing whereas the other key informants were purposively selected. Initially two meetings were held with groups of undergraduate students who were in campus at that time to introduce the study. From these meetings, a few students volunteered to participate in the study. Recruited students either recommended other students deemed to be knowledgeable on the subject matter and provided their contacts to the study team or directly reached out to potential participants and requested them to come to the study site. The process was repeated with all new interviewees until no new information was coming out of the interviews suggesting saturation was reached [48]. We determined that no new information was obtained by regularly reviewing the transcripts as the interviews were ongoing. To explore diversity, efforts were made to ensure students recommended for participation were spread across years of study, gender, program of study and region where they came from. Student leaders were preferred as they were deemed to have more insight on the subject matter. This is because as student leaders, they interacted with a large proportion of students addressing their needs, concerns and therefore likely to understand their behavioral patterns. All the 21 students invited to participate in the study gave consent and were enrolled. For the other key informants, the Dean of students and other staff working closely with students on matters SRTB were purposively recruited.

### Data collection

We conducted in-depth interviews with the key informants (students and other stakeholders). All interviews were conducted in English and were not semi-structured. Each interview lasted about an hour and was conducted at a time convenient to each key informant. Student interviews were conducted in a private room within the VCT. For convenience, stakeholder interviews were conducted in their offices. Participants were each reimbursed Kenyan shillings 350 (an equivalent to about USD 3.00) to compensate for time spent. Interviews were moderated by the main author (SC) in English and permission for notes taking and audio recording was sought *a-priori*. An interview guide was earlier developed following the World Health Organization guidelines on school-based student health surveys [49] (S1 File). Participants’ perceptions were explored using general open-ended questions followed by additional probing where appropriate. Participants’ sociodemographic data including date of birth and gender were also collected.

### Data analysis

A distribution of the study participants by their socio-demographic characteristics was done using frequencies and percentages. Audio recordings from in-depth interviews were transcribed. To ensure the study team could not directly associate the transcripts with individual participants, identifying information was not included in the transcripts. A thematic framework analysis approach [50], was applied as follows: Firstly, the transcripts were coded in QSR NVivo 12 (QSR International Ltd, Southport, UK). Initial coding was guided by major themes from the in-depth interviews and ecological model. New codes and themes were developed on the basis of the in-depth interview transcripts. Secondly, excerpts were reviewed to identify common themes and variant views. Codes representing similar themes were collapsed to develop fine codes. Finally, illustrative quotations representing each theme were presented. To develop the causal loop diagram, the ex-post approach was used [23]. First, cause and effect statements were extracted from the transcripts including the direction of the relationship (positive or negative). Next, all cause and effect statements were drawn as simple diagrams, known as causal structure diagrams (CSD [S2 File]) that included a polarity showing the direction of the relationship. Each of the CSDs were then combined to form a single CLD representing the investigators mental model. Where the data did not suggest presence of a connection between two variables of interest, extra connections were added based on literature, and where necessary variables renamed, to complete the causal system as per the investigators’ mental model. In those instances, the additional connections and variables were indicated in red. Finally, to validate the CLD, its structure was compared to the primary data used in its development [23]. Specifically, the connections between variables in the CLD, including their polarity, were compared with the transcripts to determine if they were telling the same “story.” SC prepared the CLD then held two meetings with KM to validate it. Any disagreements were discussed and consensus reached. The CLD was developed using Vensim software version 9.3.4 (Ventana systems Inc 2015).

### Ethical considerations

Prior to recruitment, a written informed consent was obtained from all potential participants. Ethical clearance was granted by Pwani University Institutional Scientific and Ethics Review Committee (ERC/PhD/003/2019) and the Kenya Medical Research Institute Scientific and Ethics Review Unit (KEMRI/SERU/CGMR-C/166/3925). Additionally, administrative approvals were granted by the National Council for Science Technology and Innovation (NACOSTI/P/19/1142).

## Results

### Characteristics of participants

In-depth interviews were conducted among students (n = 21) and other stakeholders (Pwani University staff [n = 5]). Of the 26 participants, the majority were female (n = 16 [62%]). The median age was 21 years (min/max; 18–24) and 52 years (min/max; 32–58) for students and other stakeholders, respectively (Table 1).

**Table 1:** Characteristics of emerging adults at a tertiary education institution in Coastal Kenya.

Characteristics	PU students (n = 21)	PU staff (n = 5)
	N [%]	N [%]
Median (min/max)	21 (18–24)	52 (32–58)
**Gender**		
Male	9 [43.0]	1 [20.0]
Female	12 [57.0]	4 [80.0]
**Living arrangement**		
In campus	2 [10.0]	-
Outside campus	19 [90.0]	-
**Level of study**		
Year 1	3 [14.0]	-
Year 2	7 [33.0]	-
Year 3	5 [24.0]	-
Year 4	6 [29.0]	-

### Perceived forms of SRTB among students

Overall, participants identified unprotected sex, transactional sex, cross-generational sex, multiple sex partnerships, gender-based violence, sex under influence of alcohol/drugs, early sex debut and sharing sex toys as SRTB. Unprotected sex was considered the most common form of SRTB among students. Others were transactional sex, cross-generational sex and multiple sex partnerships (Table 2).

**Table 2:** Common forms of SRTB.

SRTB	Key informants (n = 26)	Number of times SRTB described
Unprotected sex	26	101
Transactional sex	25	136
Cross generational sex	25	64
Multiple sex partners	25	67
Gender-based violence	20	51
Sex under influence of alcohol/drugs	14	21
Early sex debut	11	15
Sharing of sex toys	7	12

Participants explained that most unprotected sex occurs in the context of sex under the influence of alcohol/drugs:

“A male student… goes out with a female student. Both of them take alcohol, then they engage in the act[sex]. Of course, alcohol reduces the level of consciousness and once they have taken alcohol, they won’t even remember usage of condoms.” (Interview 1; key informant)

Participants also explained that at times students would just want to feel the pleasure of sex without the condom barrier. Where students are cohabiting, they would not see the need to use condom with their partners because they are used to each other. For students engaging in sex with older partners for financial benefits, unprotected sex would occur if the financier demanded it. At times unprotected sex was perceived to occur in the context of gender-based violence:

“Young people… don’t want to use the condom… they want to feel the pleasure.” (Interview 12; key informant)

Similarly, transactional sex was also described. Transactional sex was mostly characterized by exchange of money for sex. However, sex for grades or other favors was also described.

“Because of financial challenges will involve themselves in sexual activities with adults …. money for them to survive …they may end up contracting serious diseases.” (Interview 1; key informant)

Cross-generational sex mostly occurred in the context of “sugar mummy” and “sugar daddy” relationships where sex is exchanged for money, material things or favors including good grades.

“…the old lecturers, it is called sex for grades, where one doesn’t attend classes, she has failed, but you find in her transcript… she got an A.” (Interview 10; key informant)

Multiple sexual partnerships including concurrent partners were also considered to occur in the context of transactional sex. Participants perceived that economic hardships created the need for multiple partners.

“…Girls … they have … a boyfriend of their age and then… they look for another man who will be giving them money … mostly is an elder person, …. And this is not good because these older men don’t consider using protection…” (Interview 26; key informant)

Gender-based violence was also considered common and often took the form of forced sex and predominantly involved girls as victims. A male partner would use physical force or offer alcohol to the girl with the aim of sleeping with her when she is drunk. At times, non-alcoholic drinks laced with drugs would be offered.

“There are situations where the students engage in sex without consent from one party. So, one party is forced to accept. So, in the process they don’t even think of condoms.” (Interview 1; key informant)

“They do take advantage of ladies mostly when they go to clubs. These guys have an intention of having sex with this girl… they …give her some drug, or alcohol in excess … so they end up having sex with you, you end up getting disease, maybe HIV or STIs.” (Interview 16; key informant)

Sex under the influence of alcohol/drugs was considered risky as it was frequently unprotected.

“When people are into drugs they forget themselves and find themselves doing that act [sex] may be not protected then they can contract [STIs].” (Interview 17; key informant)

It was perceived that those engaging in SRTB are likely to have started sexual activities at an early age. Some participants felt that sexual debut could occur even among nine-year olds.

“Lady … begin early sex even … at the age of 9 years and for the men maybe 12 years.” (Interview 5; key informant)

Sharing of sex toys among lesbians was also reported and could facilitate transmission of STI including HIV.

“The lesbians…use those tools [toys]. …maybe it is a group of lesbians and maybe they are using that one vibrator and you never know if one has the STI through that they may contract.” (Interview 12; key informant)

### Risk and protective factors for SRTB among students

**Fig 1:**
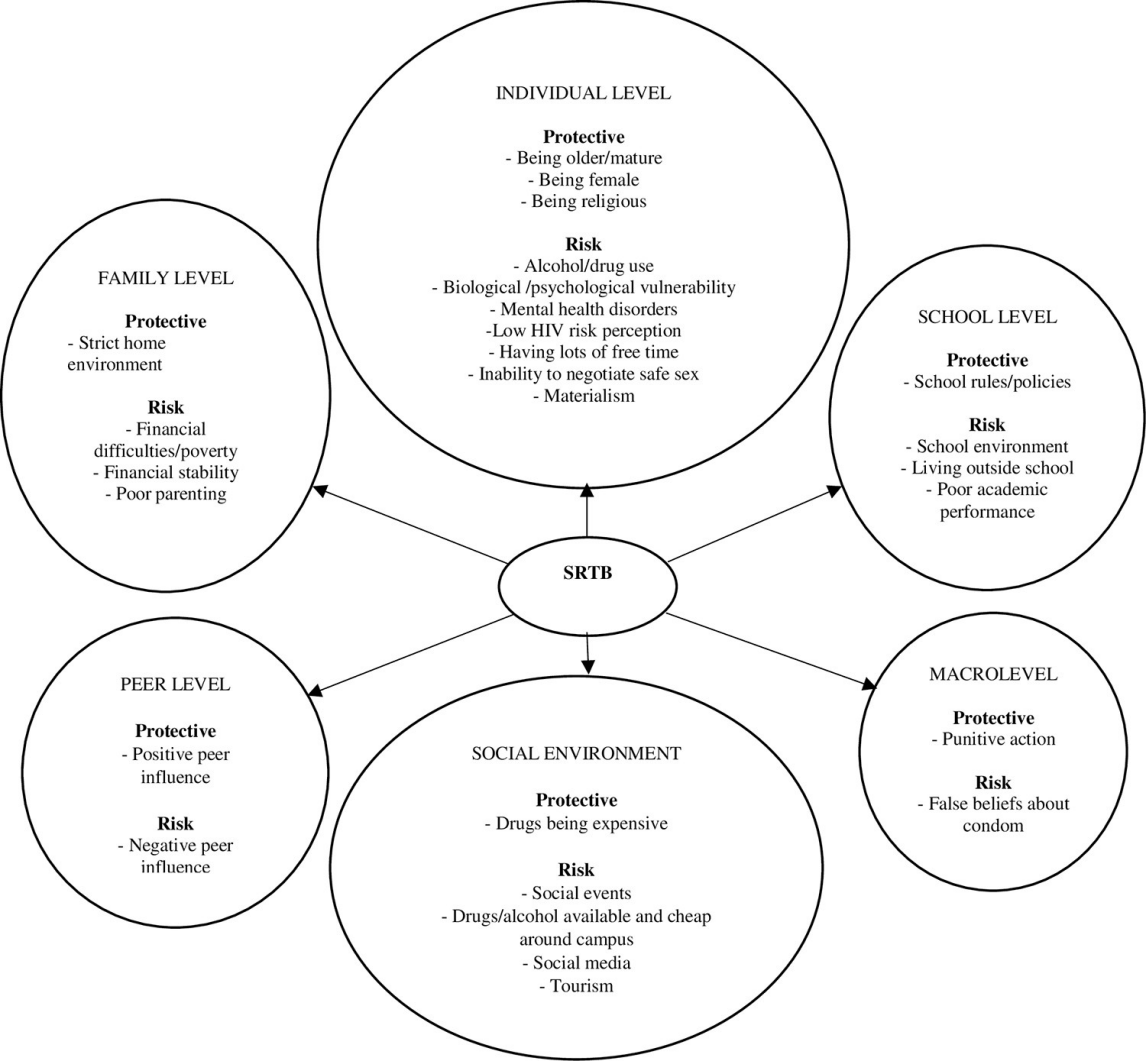
A diagram of emerging adults’ and other stakeholders’ perceived risk and protective factors for sexual risk taking behaviour among emerging adults at a tertiary education institution in Coastal Kenya.

The respondents identified a number of risk and protective factors for SRTB. Based on the ecological model, most of the reported risk factors operated at the individual level followed by those at social, family and peer level. Similarly, most of the reported protective influences operated at the individual level (Fig 1 and Table 3).

**Table 3:** Risk and protective factors of sexual risk-taking behaviour and exemplifying statements.

Factor level		Factor	Exemplifying statement
Individual	*Risk*	Alcohol/drug use	…People are normally raped because they… served juice which maybe has been laced with alcohol or whatever, in the morning they come crying …. . .for PEP [Post exposure prophylaxis] …rape has taken place and …(Interview 25; key informant).
		Biological vulnerability	…some do it[sex] for pleasure, some people who drive pleasure by having many boyfriends… they want the sex they don’t want the commitment…that is why disease and pregnancy [are common] … (Interview 14; key informant).
		Mental health disorders	…when a person is depressed they may not be in a position to … make the right decision … and they may engage more in risky behaviour, they might have reached a point where they don’t care … therefore, taking measures to have safe sex may not be in their minds in that depressed state… (Interview 23; key informant).
		Low HIV risk perception	…some don’t simply because they have a girlfriend and that girlfriend… may not want. For example, I live with a girl in my house, you know condom…becomes boring because you are with her there every now and then… but to others they may stop using simply a lady can tell you why have you decided to use a condom on me don’t you trust me. . . (Interview 20; key informant).
		Having lots of free time	…Idleness … because there is nothing you do so some will …go hung around…they date or sleep somewhere with somebody she or he doesn’t know their [HIV] status…and the end of it all you find that the person you were with may be had HIV… (Interview 5; key informant).
		Inability to negotiate safe sex	… yes, they will do it [unprotected sex] willingly…what this boy will tell you is ‘if you don’t do it then I will leave you’ then others fear they don’t have that power to refuse. (Interview 12; key informant).
		Materialism	… this occurs mostly to the girls, … if … you are greedy you see may be the money they [parents] are giving you is for food, … but you want more, … you want to live a good life … so you find that they will go look for those sponsors because those sponsors like the old men …. will do anything for you as long as you are … having sex with them… (Interview 12; key informant).
	*Protective*	Being older/mature	…the older students some of them are mature… most of them know what is good and what is bad for them but the younger students they have no limit they are not guided to know what is good and what is bad they take everything for fun (Interview 4; key informant).
		Being female	…it is not easy for a girl because …they do the sexual [activities] from the heart but the men do it for pleasure just to satisfy their desire that time and they go …so it is not easy for a girl to engage in sex easily but for the male students it is very easy … (Interview 12; key informant).
		Being religious	…A religion such as Muslim…you’ll find their religion has limited the girls to engage in sexual activities before marriage… (Interview 10; key informant).
Social environment	*Risk*	Social events	…This student decided to go for freshers’ night [party for new students]. So, when …she went home she called me and told me … I’m feeling like vomiting, I have stomach ache that is crampy. Then I told her those looks like signs of pregnancy so please go to the hospital, get a kit and test. So, when she did that the results were positive… (Interview 1; key informant).
		Drugs available & cheap in campus	…They are several, they are just around the place, they can get them very easily, and they are being sold very cheaply… (Interview 26; key informant).
		Social media	… [they find the sponsor/sugar daddy] on face book… (Interview 12; key informant).
		Tourism	You know this is coast there are rich people[tourists] around the beaches… an old guy approaches you he gives you his number “*anaanza kukukatia unaingia box*” [he seduces you and you give in] …some people can go for it because of the money (Interview 14; key informant).
	*Protective*	Drugs being expensive	Some inject but. . .they rarely do it. . . injection drugs are very expensive. . . so most of them don’t afford it. . . (Interview 10; key informant).
Peer	*Risk*	Negative peer influence	Girls especially, they are not able to make quite good decisions, and this led them to being harassed or lured into sex and latter you’ll find out they have not used protection (Interview 10; key informant).
	*Protective*	Positive peer influence	…you are talking about the same issues you can share your personal issues or you can share to the group what you went through and how you have become, it helps (Interview 14; key informant).
Family	*Risk*	Financial difficulties	Because of financial challenges will involve themselves in sexual activities with … adults to get money … to survive…. and…. end up contracting serious diseases (Interview 1; key informant).
		Financial stability	Then there is HELB money [student loan] that comes in …comrades [students] gather then they so many parties…. like parting and in those parties, alcohols are the accompaniment, sometimes in the rentals we hear them shouting… but eventually I think illicit sex takes place… (Interview 25; key informant).
		Lack of parental monitoring	That one [SRTB] will mostly depend with parental care you have at home… at an early age below 18 it is mostly influenced by the lack of proper parenting… (Interview 2; key informant).
	*Protective*	Strict home environment	When you are at home the chances of being lured [into SRTB] are very low…when your home ground is so strict… (Interview 24; key informant).
School	*Risk*	School environment	Ladies end up having sex with different guys because … she loved this guy who broke her heart, so she ends up sleeping with anyone… (Interview 16; key informant).
		Living outside school	If they are out there [residence outside campus] …. they have the freedom… then it means they can involve in anything and as youth we know there are many challenges particularly on alcohol and drug abuse, matters of sexual behaviour… (Interview 22; key informant).
		Poor academic performance	…they make…poor judgment in their academics this results to their failure and causes them to engage in sexual behaviour which are risky… (Interview 10; key informant).
	*Protective*	School policies/rules	…the university issue condoms to all students free… (Interview 25; key informant).
Macro	*Risk*	False belief about condom	There is that notion that when you put on a condom, the sexual effect is not as sweet as when you are not using… (Interview 24; key informant).
	*Protective*	Punitive action	In campus you will face the consequences definitely you would like to engage [in SRTB] out there… (Interview 23; key informant).

The causal loop diagram showed the interconnectedness of the factors contributing to SRTB as illustrated by the numerous feedback mechanisms that influence SRTB. Transactional sex, cross generational sex and multiple sex partnerships formed a reinforcing feedback loop [R2] where each had an incremental effect on the other. The loop was driven by sex tourism, visibility of sugar mummies/daddies on social media, financial difficulties, poor academic performance, liberal sexual norms and the desire to acquire material things. However, being mature/older age and being female reduced transactional sex and multiple sex partners respectively, which consequently had the same effect on all components of the loop.

**Fig 2:**
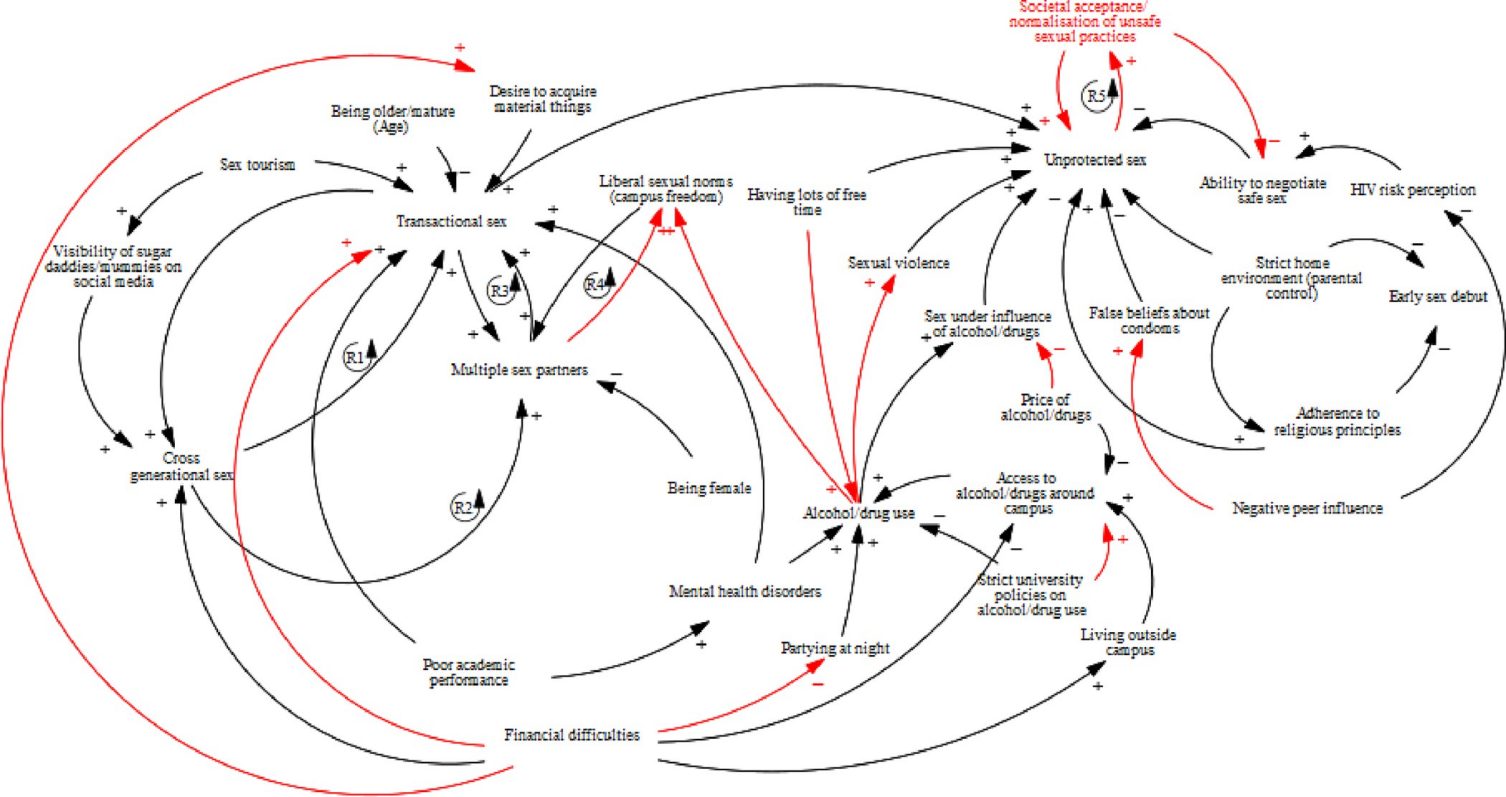
A causal loop diagram showing risk and protective factors that impact sexual risk-taking behaviour among emerging adults at a tertiary education institution in Coastal Kenya. Arrows and variables in red have been added based on literature to make the causal loop diagram complete. R- Reinforcing loop.

Similarly, societal acceptance of unsafe sexual practices seemed to increase unprotected sex and formed a reinforcing loop with it [R5]. Transactional sex, having lots of free time, sexual violence, sex under influence of alcohol/drugs and false beliefs about condom increased unprotected sex therefore introducing an incremental effect on the reinforcing loop between unprotected sex and societal acceptance of unsafe sex [R5]. However, adherence to religious principles, parental control and ability to negotiate safe sex reduced unprotected sex. (Fig 2).

### Individual level factor

*Risk factors*. *Alcohol/drug use*: Participants explained that alcohol/drug use was a contributing factor to SRTB. In some instances, male students would buy alcohol for female students with the intention of engaging in sex with them once they get drunk. In these circumstances the male partner is taking advantage of the drunk state of the girl. Where the girl is still conscious enough to resist, the male partner would use force. Participants also narrated that at times the intention to take advantage of one partner was absent but sexual risk behaviour would still happen because alcohol impedes their decision making. In addition, it was revealed that risky sexual behaviors were happening after abusing drugs other than alcohol.

“People are normally raped because they… served juice which maybe has been laced with alcohol …, in the morning they come … for PEP [Post exposure prophylaxis] …rape has taken place.” (Interview 25; key informant).

“When somebody is drunk … they cannot decide very well, even if they wanted to use a condom …, they will not use it.” (Interview 26; key informant).

*Biological/psychological vulnerability*: Participants felt that biological factors including sex and personal characteristics like being inclined to have fun, curiosity/wanting to explore and wanting to be famous were contributing to SRTB by emerging adults. Interestingly, both male and female sex were reported to increase vulnerability to SRTB though most participants were of the view that being male was a contributing factor to SRTB.

“…some do it[sex] for pleasure, some people who drive pleasure by having many boyfriends… they want the sex they don’t want the commitment…that is why disease and pregnancy [are common].” (Interview 14; key informant).

*Mental health disorders*: Mental health disorders especially depression and anxiety were perceived to be pushing emerging adults to engage in alcohol and subsequently SRTB.

“…when a person is depressed they may not be in a position to … make the right decision … and they may engage more in risky behaviour, they might have reached a point where they don’t care … therefore, taking measures to have safe sex may not be in their minds in that depressed state.” (Interview 23; key informant).

*Low HIV risk perception*: Participants reported that emerging adults who believe have low chances of contracting HIV were more likely to engage in SRTB. Participants reported that emerging adults would engage in sex without condom because they do not like using it, have negative perception about it or they are used to their partner so do not see the need to use condom. Further, low HIV risk perception among emerging adults was exemplified by other risky activities/beliefs that they were reported to engage in including deep kissing and fear of pregnancy but not HIV infection.

“…some don’t simply because they have a girlfriend and that girlfriend… may not want. For example, I live with a girl in my house, you know condom…becomes boring because you are with her there every now and then… but to others they may stop using simply a lady can tell you why have you decided to use a condom on me don’t you trust me?” (Interview 20; key informant).

“… lips [deep] kissing can lead to contacting of HIV” (Interview 4; key informant).

*Having a lot of free time and inability to negotiate safe sex*: These were also seen to contribute to SRTB. Participants explained that students with a lot of free time tend to spend that time thinking about and engaging in SRTB.

“Idleness … because there is nothing you do so some will …go hung around…they date or sleep somewhere with somebody she or he doesn’t know their [HIV] status…and the end of it all you find that the person you were with may be had HIV.” (Interview 5; key informant).

“Yes, they will do it [unprotected sex] willingly…what this boy will tell you is ‘if you don’t do it then I will leave you’ then others fear they don’t have that power to refuse.” (Interview 12; key informant).

*Materialism*: Participants revealed that the love for money and material things was pushing especially women into transactional sex just to maintain their desired lifestyle.

“… this occurs mostly to the girls, … if … you are greedy you see may be the money they [parents] are giving you is for food, … but you want more, … you want to live a good life … so you find that they will go look for those sponsors because those sponsors like the old men … will do anything for you as long as you are … having sex with them” (Interview 12; key informant).

*Protective factors*. *Being older/mature*: Participants explained that unlike the younger/junior students, mature students are focused on future life and academics and generally know what is good for them so they are more likely to be careful when making decisions regarding sexual matters.

“The older students some of them are mature… most of them know what is good and what is bad for them but the younger students they have no limit they are not guided to know what is good and what is bad they take everything for fun.” (Interview 4; key informant).

*Being female*: It was suggested that males are more prone to careless sex because they do it for pleasure with no emotional attachment unlike women.

“It is not easy for a girl because …they do the sexual [activities] from the heart but the men do it for pleasure just to satisfy their desire that time and they go …so it is not easy for a girl to engage in sex easily but for the male students it is very easy.” (Interview 12; key informant).

*Being religious*: Religion was perceived to help reduce SRTB since most religions prohibit sex before marriage.

“A religion such as Muslim…you’ll find their religion has limited the girls to engage in sexual activities before marriage.” (Interview 10; key informant).

*Social environment factors*. *Risk factors*. *Social events*: Participants narrated that social events organized both inside and outside campus provided an opportunity for students to engage in SRTB. Freedom enjoyed by the students especially outside campus was perceived to facilitate SRTB during the social events.

“This student decided to go for freshers’ night [party for new students]. So, when …she went home she called me and told me … I’m feeling like vomiting, I have stomach ache that is crampy. Then I told her those looks like signs of pregnancy so please go to the hospital, get a kit and test. So, when she did that the results were positive.” (Interview 1; key informant).

*Drugs/alcohol available and cheap around campus*: Participants felt that alcohol and other drugs were readily available and cheap around campus which increased the risk of engaging in sex while under influence of the drugs.

“They are several, they are just around the place, they can get them very easily, and they are being sold very cheaply.” (Interview 26; key informant).

*Social media*: Social media use was seen to predispose students to SRTB. Participants narrated how students connect with their sugar mummies/daddies on Facebook. Further, they explained that students watch pornography which fuels their curiosity to try it out.

“[they find the sponsor/sugar daddy] on face book.” (Interview 12; key informant).

*Tourism*: It was perceived that tourism contributed to SRTB. Since the university is located along the Kenyan Coast, some tourists, usually old, along the beaches were targeting female students. The students would in turn give in as they expect to benefit financially.

“You know this is Coast there are rich people[tourists] around the beaches… an old guy approaches you, he gives you his number “*anaanza kukukatia unaingia box*” [he seduces you and you give in] …some people can go for it because of the money.” (Interview 14; key informant).

*Protective factors*. *Drugs being expensive*: While participants felt that drugs were easily accessible and cheap, especially palm wine, locally known as “*mnazi*”, there were also expensive drugs that students could not access. This keeps them sober hence reducing the chances of engaging in SRTB.

“Some inject but …they rarely do it… injection drugs are very expensive… so most of them don’t afford it…” (Interview 10; key informant).

### Peer level factors

*Risk factors*. *Negative peer influence*: Most participants felt that close friends can influence others into risky sexual encounters. Some of the negative influences include non-condom use during a sexual encounter while drunk.

“Girls especially, they are not able to make quite good decisions, and this led them to being harassed or lured into sex [due to negative peer influence] and latter you’ll find out they have not used protection.” (Interview 10; key informant).

### Protective factors

*Positive peer influence*: Sharing experiences by peers was seen to help them overcome common challenges.

“… you are talking about the same issues you can share your personal issues or you can share to the group what you went through and how you have become, it helps.” (Interview 14; key informant).

*Family level factors*. *Risk factors*. *Financial difficulties/poverty*: Participants narrated that due to financial challenges, students would engage in SRTB in order to cater for their needs.

“Because of financial challenges will involve themselves in sexual activities with … adults to get money … to survive…. and…. end up contracting serious diseases.” (Interview 1; key informant).

*Financial stability*: Though financial difficulties were perceived to predispose to SRTB, financial stability was equally perceived to have the same effect. Financially stable students or those with well-off friends were more predisposed to SRTB because they could afford the expenses associated with such activities including going out for social events with their partners.

“Then there is HELB money [student loan] that comes in …comrades [students] gather then they so many parties…. like parting and in those parties, alcohols are the accompaniment, sometimes in the rentals we hear them shouting… but eventually I think illicit sex takes place.” (Interview 25; key informant).

*Lack of parental monitoring*: Decision making on sexual matters was seen to be influenced by parental care that one receives at an early age. Lack of close parental monitoring was seen to negatively influence decision making regarding sexuality leading to SRTB.

“That one [SRTB] will mostly depend with parental care you have at home… at an early age below 18 it is mostly influenced by the lack of proper parenting.” (Interview 2; key informant).

*Protective factors*. *Strict home environment*: A strict home environment where there is close parental monitoring was perceived to offer protection from being lured into SRTB.

“When you are at home the chances of being lured [into SRTB] are very low…when your home ground is so strict.” (Interview 24; key informant).

### School level factors

*Risk factors*. *School environment*: Participants explained that some factors within the school environment could predispose students to SRTB. Most participants pointed out that sexual relationships in campus are temporary and sometimes students find themselves engaging in SRTB to cope with heart break following break-up of a relationship. Similarly, campus freedom that allows students to have sexual relationships and weak security systems at campus were blamed for SRTB among students.

“Ladies end up having sex with different guys because … she loved this guy who broke her heart, so she ends up sleeping with anyone.” (Interview 16; key informant).

*Living outside school*: Compared to residence within campus, participants were of the view that residing outside campus exposed students to SRTB. This is because the campus freedoms are there but are practiced within the rules in campus. This is not the case outside campus where the campus rules do not apply.

“If they are out there [residence outside campus] …. they have the freedom… then it means they can involve in anything and as youth we know there are many challenges particularly on alcohol and drug abuse, matters of sexual behaviour.” (Interview 22; key informant).

*Poor academic performance*: Participants explained that when students perform poorly in academics, some get depressed and end up engaging in SRTB. Female students performing poorly would be ready to have risky sexual relations with lecturers to rescue themselves.

“… they make…poor judgment in their academics this results to their failure and causes them to engage in sexual behaviour which are risky.” (Interview 10; key informant).

*Protective factors*. School rules or policies: Participants felt that school policies and rules which directly affects those living in campus stops students from engaging in SRTB. Examples of policies include restriction of alcohol use and provision of mental health counselling services on campus which keeps frustrated students from engaging in SRTB as a way of coping. Similarly, provision of condom on campus was mentioned.

“… the university issues condoms to all students free.” (Interview 25; key informant).

### Macro level factors

*Risk factors*. *False beliefs about condoms*: Participants explained that some students were not using condom because of certain beliefs. Some believed that condoms reduce sexual pleasure while others believe condoms will burst.

“There is that notion that when you put on a condom, the sexual effect is not as sweet as when you are not using…” (Interview 24; key informant).

*Protective factors*. *Punitive action*: It was revealed that negative consequences imposed on students for engaging in SRTB makes them shy away from it. Participants explained that students caught engaging in sex in public spaces within campus due to influence of alcohol face consequences including expulsion from the university.

“In campus you will face the consequences definitely you would like to engage [in SRTB] out there.” (Interview 23; key informant).

## Discussion

In this study we aimed to understand the perceived SRTBs among emerging adults and associated risk factors. Our findings show that emerging adults and other stakeholders perceived SRTB to be common at an institution of higher learning in Coastal Kenya, especially unprotected sex, transactional sex, cross generational sex, multiple sexual partnerships, gender-based violence, sex under influence of alcohol/drugs, early sex debut, and sharing sex toys. Biological/psychological vulnerability, negative peer influence, alcohol/drug use and financial difficulties were most frequently described as drivers of SRTB in this population.

Unprotected sex, sex under the influence of alcohol/drugs and gender-based violence were described to be common among the emerging adults. Further, the causal loop diagram suggests that the three are interlinked with gender-based violence and sex under influence of alcohol/drugs increasing unprotected sex. Consistent with our findings, previous studies from Sub-Saharan Africa have found unprotected sex to be the most common SRTB which underscores its importance [14]. Indeed, a recent systematic review and meta-analysis of emerging adults age 18–25 years from Africa found that non-condom use had the highest pooled prevalence estimate of 46% [14]. Participants explained that alcohol/drugs impair judgement hence when engaging in sex while under influence, emerging adults may not bother using a condom and if they do, it will not be used correctly. In addition, gender-based violence seems to be fueled by alcohol/drug use in this population. Social events organized both on and off campus provide an opportunity for alcohol/drug use. In these circumstances, perpetrators of gender-based violence would offer alcohol/drugs to the targeted victims then assault them sexually. It is possible that alcohol/drug use and the social events could be behind unprotected sex, sex under influence of alcohol and gender-based violence in this population as it has been found in other studies [14]. The causal loop diagram similarly suggests that alcohol/drug use increases sex under influence of alcohol/drugs which in turn increases unprotected sex. There is need for universities to strengthen mental health support services including counselling to help reduce alcohol/drug use among emerging adults [51].

Transactional sex, cross generational sex and multiple sex partners were perceived to be common amongst emerging adults and mainly occurred in the context of sex in exchange of money. Further, the causal loop diagram shows that the three SRTBs are interlinked in a reinforcing loop where transactional sex increases cross generational sex which then increases multiple sex partnerships that further increases transactional sex in a vicious cycle [R2]. A recent review equally found transactional sex, cross-generational sex and multiple sex partnerships to be prevalent among emerging adults in Sub-Saharan Africa [14]. Similarly, financial difficulties came out strongly among participants as a risk factor for transactional sex. The Kenyan government has been implementing the 100% transition policy which aims to ensure all school children progress and finally either join university or middle level colleges [52,53]. This may have contributed to the increase in public university enrollment from 442,000 in 2015 to 1,000,000 in 2018 [47]. Interestingly, government funding to public universities has reduced [47]. Public universities have had to bridge the funding gap by passing certain costs to parents/students. Additionally, students’ financial burden has further been increased by the fact that many of them miss out on government loans to fund their education due to their increasing numbers [54]. Students welfare has also posed a challenge as most students are accommodated outside campus therefore paying rent at market price. It is possible that the fore mentioned factors may have rendered university education expensive hence parents struggle to support their children. It is plausible that some students engage in transactional sex, cross generational sex and multiple sex partnerships to cater for their needs at the university. However, materialism and peer pressure were also described to be risk factors for SRTB. It is possible that the desire to acquire material things to maintain an expensive life style like their financially-stable peers was pushing emerging adults to engage in transactional sex, cross generational sex and multiple sex partnerships like it has been reported in previous studies [55]. The causal loop diagram equally suggests that financial difficulties and desire to acquire material things directly increase transactional sex, cross generational sex and multiple sex partnerships. Kenyan universities need to enhance sexual education programs for students so that they can be empowered and avoid the urge to engage in SRTB for financial gain [56]. Importantly, the Kenyan government is currently reviewing the funding model for public universities with a view to bridging the funding short fall [57]. Although transactional sex mainly involved exchanging sex for money, sex for favorable grades was also reported. A previous study among adolescents in this setting equally revealed that transactional sex was common but it did not involve exchange of sex for favorable grades despite the fact that some of the adolescents were school-going [55]. It seems exchange of sex for favorable grades is a common form of transactional sex among young people in universities but not in high schools or primary schools. Kenyan universities have administrative procedures to deal with sex for grades. It is probably time for the universities to review those procedures and strengthen them to ensure such cases are reduced.

One strength of this study is the use of causal loop diagram to map the SRTB system in this population. An important limitation of this study is that it only involved participants from one university. It remains unclear if their views could differ from those in other universities given the geographical differences.

## Conclusion

This study explored SRTB among emerging adults at a public university in Coastal Kenya. Consistent with findings from studies among emerging adults in general population, our study shows that emerging adults are engaging in SRTB with unprotected sex perceived to be the most common and seems to be driven by individual level factors especially alcohol/drug use. Uniquely, our study utilizes systems thinking to build on existing literature by highlighting the perceived drivers of SRTB among university students and their interlinkages. Enhancing sexual education programs for university students in Kenya and strengthening support systems including counselling for those using alcohol/drugs may help reduce SRTB among emerging adults in Kenyan universities.

## Supporting information

S1 File Interview guide. (DOCX)

S2 File Causal structure diagram. (DOCX)

S3 File STROBE statement—checklist of items that should be included in reports of observational studies. (DOCX)

## References

[1] Gibbs A, Hatcher A, Jewkes R, Sikweyiya Y, Washington L, Dunkle K, et al. Associations between Lifetime Traumatic Experiences and HIV-Risk Behaviors among Young Men Living in Informal Settlements in South Africa: A Cross-Sectional Analysis and Structural Equation Model. Journal of Acquired Immune Deficiency Syndromes. 2019;81(2):193–201. doi: 10.1097/QAI.0000000000002010 30893127 PMC6553984

[2] Knox J, Reddy V, Lane T, Lovasi GS, Hasin D, Sandfort T. Safer sex intentions modify the relationship between substance use and sexual risk behavior among black South African men who have sex with men. International Journal of STD and AIDS. 2019;30(8):786–94. doi: 10.1177/0956462418825333 31142222 PMC6765215

[3] Masa R, Graham L, Khan Z, Chowa G, Patel L. Food insecurity, sexual risk taking, and sexual victimization in Ghanaian adolescents and young South African adults. International journal of public health. 2019;64(2):153–63. doi: 10.1007/s00038-018-1155-x 30105507

[4] Price J, Pettifor A, Selin A, Wagner RG, MacPhail C, Agyei Y, et al. The association between perceived household educational support and HIV risk in young women in a rural South African community (HPTN 068): A cross sectional study. PLoS ONE. 2019;14(1):e0210632. doi: 10.1371/journal.pone.0210632 30653540 PMC6336295

[5] Tarkang EE, Pencille LB, Dadah E, Nzegge MM, Komesuor J. Highly prevalent at-risk sexual behaviours among out-of-school youths in urban Cameroon. The Pan African medical journal. 2018;30:254. Epub 2019/01/15. doi: 10.11604/pamj.2018.30.254.15775 ; PubMed Central PMCID: PMC:6317297. 30637039 PMC6317297

[6] Yamanis TJ, Fisher JC, Moody JW, Kajula LJ. Young Men’s Social Network Characteristics and Associations with Sexual Partnership Concurrency in Tanzania. AIDS and behavior. 2016;20(6):1244–55. doi: 10.1007/s10461-015-1152-5 26271813 PMC4753135

[7] Ziraba A, Orindi B, Muuo S, Floyd S, Birdthistle IJ, Mumah J, et al. Understanding HIV risks among adolescent girls and young women in informal settlements of Nairobi, Kenya: Lessons for DREAMS. PLoS ONE. 2018;13(5):e0197479. doi: 10.1371/journal.pone.0197479 29851988 PMC5978990

[8] VanderEnde K, Chiang L, Mercy J, Shawa M, Hamela J, Maksud N, et al. Adverse Childhood Experiences and HIV Sexual Risk-Taking Behaviors Among Young Adults in Malawi. Journal of interpersonal violence. 2018;33(11):1710–30. Epub 2018/05/10. doi: 10.1177/0886260517752153 ; PubMed Central PMCID: PMC:6158791. 29739289 PMC6158791

[9] Reynolds Z, Gottert A, Luben E, Mamba B, Shabangu P, Dlamini N, et al. Who are the male partners of adolescent girls and young women in Swaziland? Analysis of survey data from community venues across 19 DREAMS districts. PLoS ONE. 2018;13(9):e0203208. doi: 10.1371/journal.pone.0203208 30216356 PMC6157821

[10] Ware E, Tura G, Alemu T, Andarge E. Disparities in risky sexual behavior among khat chewer and non- chewer college students in Southern Ethiopia: a comparative cross-sectional study. BMC public health. 2018;18(1):558. doi: 10.1186/s12889-018-5405-x 29703181 PMC5921970

[11] Faini D, Hanson C, Baisley K, Kapiga S, Hayes R. Sexual behaviour, changes in sexual behaviour and associated factors among women at high risk of HIV participating in feasibility studies for prevention trials in Tanzania. PLoS ONE. 2020;15(4):e0231766. doi: 10.1371/journal.pone.0231766 32298383 PMC7162511

[12] Mmbaga EJ, Leyna GH, Leshabari MT, Moen K. Early Anal Sex Experience Among Men Who Have Sex with Men in Dar Es Salaam Tanzania: Implications for HIV Prevention and Care. Archives of sexual behavior. 2019. 10.1007/s10508-019-01529-5. 31872388

[13] Stoebenau K, Nair RC, Rambeloson V, Rakotoarison PG, Razafintsalama V, Labonte R. Consuming sex: The association between modern goods, lifestyles and sexual behaviour among youth in Madagascar. Globalization and Health. 2013;9(1):13. 10.1186/1744-8603-9-13. 23510104 PMC3651287

[14] Chea SK, Kagonya VA, Abdullahi O, Abubakar AA, Abbeddou S, Michielsen K, et al. Sexual risk-taking behavior amongst emerging adults in Africa: a systematic review and meta-analysis. medRxiv. 2022:2022.09.13.22279893 [Preprint]. Available from: 10.1101/2022.09.13.22279893. 38895966

[15] UNAIDS. UNAIDS DATA 2020. Geneva, Switzerland: United Nations, 2020.

[16] NASCOP. 2020 World AIDS day report. Kenya HIV progress indicators. Nairobi: National AIDS and STI Control Program, 2020.

[17] Levinson D. The seasons of a man’s life. New York: Ballantine books, an imprint of the Random House Publishing Group, a division of random House Inc, New York; 1978.

[18] Arnett JJ. Emerging adulthood: A theory of development from the late teens through the twenties. American Psychologist. 2000;55(5):469–80. 10.1037//0003-066x.55.5.469. 10842426

[19] NACC. Kilifi County HIV and AIDS strategic plan 2016–2020. National AIDS Control Council, 2016.

[20] NCRC. Gender based violence in Kenya. Nairobi: National Crime Research Centre., 2014.

[21] Blum Rw Fau—McNeely C, McNeely C Fau—Nonnemaker J, Nonnemaker J. Vulnerability, risk, and protection. J Adolesc Health. 2002;31(1054-139X (Print)). doi: 10.1016/s1054-139x(02)00411-1 . 12093609

[22] Rusoja EA-O, Haynie DA-O, Sievers JA-O, Mustafee NA-O, Nelson FA-O, Reynolds MA-O, et al. Thinking about complexity in health: A systematic review of the key systems thinking and complexity ideas in health. J Eval Clin Pract. 2018;24(3):600–6. doi: 10.1111/jep.12856 PubMed Central PMCID: PMC. 29380477

[23] Cassidy R, Borghi J, Semwanga AR, Binyaruka P, Singh NS, Blanchet K. How to do (or not to do)…using causal loop diagrams for health system research in low and middle-income settings. Health Policy and Planning. 2022;00(00):1–9. 10.1093/heapol/czac064. PMC9661310 35921232

[24] Zhang J, Tang BW, Liu MW, Yuan S, Yu HJ, Zhang R, et al. Association of Adverse Childhood Experiences with Health Risk Behaviors Among College Students in Zambia. International journal of behavioral medicine. 2020. doi: 10.1007/s12529-020-09863-y 32096097

[25] Gebresllasie F, Tsadik M, Berhane E. Potential predictors of risk sexual behavior among private college students in Mekelle City, North Ethiopia. PLoS One. 2017;28:151. Epub 2018/06/01. doi: 10.11604/pamj.2017.28.151.5370 ; PubMed Central PMCID: PMC:5978990. 29564034 PMC5851670

[26] Hoffman S, Levasseur M, Mantell JE, Beksinska M, Mabude Z, Ngoloyi C, et al. Sexual and reproductive health risk behaviours among South African university students: results from a representative campus-wide survey. African journal of AIDS research: AJAR. 2017;16(1):1–10. Epub 2017/04/04. doi: 10.2989/16085906.2016.1259171 ; PubMed Central PMCID: PMC:5563261. 28367750 PMC5563261

[27] Bulled NL. Social models of HIV risk among young adults in Lesotho. African Journal of AIDS Research. 2015;14(3):239–54. 10.2989/16085906.2015.1054295. 26284999

[28] Othieno CJ, Okoth R, Peltzer K, Pengpid S, Malla LO. Risky HIV sexual behaviour and depression among University of Nairobi students. Annals of General Psychiatry. 2015;14(1):16. doi: 10.1186/s12991-015-0054-2 25873984 PMC4396741

[29] Agardh A, Cantor-Graae E, Ostergren P-O. Youth, sexual risk-taking behavior, and mental health: A study of university students in Uganda. International Journal of Behavioral Medicine. 2012;19(2):208–16. doi: 10.1007/s12529-011-9159-4 21590465 PMC3358553

[30] Choudhry V, Agardh A, Stafstrom M, Ostergren PO. Patterns of alcohol consumption and risky sexual behavior: a cross-sectional study among Ugandan university students. BMC public health. 2014;14:128. doi: 10.1186/1471-2458-14-128 24502331 PMC3933239

[31] Dingeta T, Oljira L, Assefa N. Patterns of sexual risk behavior among undergraduate university students in Ethiopia: a cross-sectional study. The Pan African medical journal. 2012;12:33. Epub 2012/08/15. ; PubMed Central PMCID: PMC:3415054. 22891091 PMC3415054

[32] Imaledo JA, Peter-Kio OB, Asuquo EO. Pattern of risky sexual behavior and associated factors among undergraduate students of the University of Port Harcourt, Rivers State, Nigeria. The Pan African medical journal. 2012;12:97. 23133697 PMC3489398

[33] Mehra D, Kyagaba E, Ostergren PO, Agardh A. Association between self-reported academic performance and risky sexual behavior among Ugandan university students- a cross sectional study. Global journal of health science. 2014;6(4):183–95. doi: 10.5539/gjhs.v6n4p183 24999121 PMC4825383

[34] Mulu W, Yimer M, Abera B. Sexual behaviours and associated factors among students at Bahir Dar University: a cross sectional study. Reproductive health. 2014;11:84. doi: 10.1186/1742-4755-11-84 25481831 PMC4271440

[35] Peltzer K, Pengpid S. Mental health correlates of HIV risk behaviour and STIs/HIV infection among university students from 22 low, middle and high income countries. Journal of Psychology in Africa. 2015;25(2):121–6.

[36] Peltzer K, Pengpid S, Tiembre I. Mental health, childhood abuse and HIV sexual risk behaviour among university students in Ivory Coast. Annals of General Psychiatry. 2013;12. 10.1186/1744-859X-12-18. 23758850 PMC3682872

[37] Salawu AT, Reis SO, Fawole OI, Dairo MD. Sexual behaviour and use of electronic media among undergraduates in the University of Ibadan. African journal of medicine and medical sciences. 2015;44(4):321–7. 27462694

[38] Samuel L, Angamo MT. Substance use and sexual risk behavior and factors associated with HIV transmission in Southern Ethiopia. International Journal of Pharmaceutical Sciences and Research. 2012;3(4):1080–6.

[39] Wasie B, Belyhun Y, Moges B, Amare B. Effect of emergency oral contraceptive use on condom utilization and sexual risk taking behaviours among university students, Northwest Ethiopia: a cross-sectional study. BMC research notes. 2012;5:501. Epub 2012/09/14. PubMed Central PMCID: PMC:3494538. doi: 10.1186/1756-0500-5-501 22971668 PMC3494538

[40] Pengpid S, Peltzer K, Skaal L. Mental health and HIV sexual risk behaviour among University of Limpopo students. South African Journal of Psychiatry. 2013;19(2):25–30. 10.7196/SAJP.415.

[41] Somba MJ, Mbonile M, Obure J, Mahande MJ. Sexual behaviour, contraceptive knowledge and use among female undergraduates’ students of Muhimbili and Dar es Salaam Universities, Tanzania: a cross-sectional study. BMC women’s health. 2014;14:94. Epub 2014/08/08. doi: 10.1186/1472-6874-14-94 ; PubMed Central PMCID: PMC:4126911. 25099502 PMC4126911

[42] Oye-Adeniran BA, Aina OF, Gbadegesin A, Ekanem EE. Substance use and sexual behaviour among female students in Nigerian universities. International quarterly of community health education. 2014;35(1):73–83. Epub 2014/11/25. doi: 10.2190/IQ.35.1.f . 25416433

[43] Ngatu NR, Hirota R, Eitoku M, Muzembo BA, Nishimori M, Kuramochi M, et al. Perception of the risk of sexual transmission of HIV among Congolese and Japanese university students. Environmental Health and Preventive Medicine. 2012;17(2):139–46. doi: 10.1007/s12199-011-0232-z 21861117 PMC3342628

[44] Pence BW, Whetten K, Shirey KG, Yao J, Thielman NM, Whetten R, et al. Factors associated with change in sexual transmission risk behavior over 3 years among HIV-infected patients in Tanzania. PLoS ONE. 2013;8(12). doi: 10.1371/journal.pone.0082974 24367575 PMC3867466

[45] Ahmed ZD, Ibrahim BS, Bolaji ML, Mohammed Y, Nguku P. Knowledge and utilization of contraceptive devices among unmarried undergraduate students ofa tertiary institution in Kano State, Nigeria 2016. Journal of Obstetrics and Gynaecology Research. 2017;43(Supplement 1):137. 10.1111/jog.13393. PMC5409995 28491234

[46] Peltzer K, Jones D, Weiss SM, Villar-Loubet O, Shikwane E. Sexual risk, serostatus and intimate partner violence among couples during pregnancy in rural South Africa. AIDS and Behavior. 2013;17(2):508–16. doi: 10.1007/s10461-012-0185-2 22488126 PMC4721242

[47] CUE. University statistics 2017/2018. Commission for University Education, 2018.

[48] Saunders B, Sim J, Kingstone T, Baker S, Waterfield J, Bartlam B, et al. Saturation in qualitative research: exploring its conceptualization and operationalization. Qual Quant. 2018;52(4):1893–907. Epub 2018/06/26. PubMed Central PMCID: PMC: 5993836. doi: 10.1007/s11135-017-0574-8 29937585 PMC5993836

[49] WHO. Global school-based student health survey 2021 version Geneva: World Health Organization, 2015.

[50] Smith J, Firth J. Qualitative data analysis: the framework approach. Nurse researcher. 2011;18(2):52–62. doi: 10.7748/nr2011.01.18.2.52.c8284 21319484

[51] Patton R, Deluca P Fau—Kaner E, Kaner E Fau—Newbury-Birch D, Newbury-Birch D Fau—Phillips T, Phillips T Fau—Drummond C, Drummond C. Alcohol screening and brief intervention for adolescents: the how, what and where of reducing alcohol consumption and related harm among young people. Alcohol Alcohol. 2014;49(2):207–12(1464–3502 (Electronic)). doi: 10.1093/alcalc/agt165 PubMed Central PMCID: PMC:3932830. 24232178 PMC3932830

[52] MoEST. Kenyan education sector support program 2005–2010. Nairobi: Ministry of Education Science and Technology, Kenya, 2005.

[53] Otieno MA, Ochieng JA. Impact of 100 Per Cent Transition Policy on Public Secondary Schools in Machakos Sub- County, Kenya: Focusing on Coping Strategies. Journal of Education and Practice. 2020;24.

[54] HELB. The 2020/2021 HELB sustainability report. Higher Education Loans Board, 2021.

[55] Ssewanyana D, Mwangala PN, Marsh V, Jao I, van Baar A, Newton CR, et al. Young people’s and stakeholders’ perspectives of adolescent sexual risk behavior in Kilifi County, Kenya: A qualitative study. Journal of Health Psychology. 2018;23(2):188–205. doi: 10.1177/1359105317736783 PubMed Central PMCID: PMC29076401. 29076401 PMC5772428

[56] Gómez-Lugo M, Morales A, Saavedra-Roa A, Niebles-Charris J, Abello-Luque D, Marchal-Bertrand L, et al. Effects of a Sexual Risk-Reduction Intervention for Teenagers: A Cluster-Randomized Control Trial. AIDS and Behavior. 2022;26(7):2446–58. doi: 10.1007/s10461-022-03574-z 35084613 PMC9162964

[57] Government of Kenya. The Kenya Gazette Nairobi: Government printer; 2022 [cited Vol. CXXIV–No. 202 Gazette notice No. 11920]. Available from: https://nation.africa/resource/blob/3968482/afef6c819d44e8605b3f501ace93e31e/cbc-gazette-notice-data.pdf.

